# Regional variations in serum pepsinogen levels and their influencing factors: a multi-center cross-sectional study

**DOI:** 10.1038/s41598-026-38326-9

**Published:** 2026-02-08

**Authors:** Huang Jiaojiao, Yu Jiaying, Tong Yuling

**Affiliations:** Department of General Practice/Health Management Center, School of Medicine, The Second Affiliated Hospital of Zhejiang University, Hangzhou, 310000 China

**Keywords:** Serum pepsinogen, Baseline level, Regional heterogeneity, Cross-sectional study, Influencing factors, Biomarkers, Cancer, Diseases, Gastroenterology, Medical research

## Abstract

Serum pepsinogen (PG) levels are recognized biomarkers influenced by various factors, including lifestyle, *Helicobacter pylori* (*H. pylori*) infection status, and the presence of gastric mucosal lesions. However, whether baseline serum PG levels exhibit regional heterogeneity independent of concurrent gastric mucosal lesions has not been clearly established. The study aims to investigate the variability of baseline serum PG levels and identify potential influencing factors across China. Data were collected from individuals undergoing routine health checkups at twelve collaborating medical centers across China between October 2016 and October 2021. Serum pepsinogen I (PGI) and pepsinogen II (PGII) were measured and the pepsinogen I/II ratio (PGR) was calculated; gastroscopy with histopathology was performed to define gastric mucosal status. Detection of *H. pylori* infection status was performed using histology, serology, and/or breath tests. Lifestyle factors were obtained via standardized questionnaires. Regional differences and associated factors were evaluated using t-tests/ANOVA and multivariable linear regression (Stata 18). A total of 2902 individuals were included in the study. Of them, 2382 was classified as non-atrophic gastritis (NAG), and their regional distribution varied. Baseline serum PG levels significantly varied by regions, except between Southern and Central China. Factors such as *H. pylori*-positive (including prior eradication), high salt intake, and frequent fruit consumption were significantly associated with baseline serum PGR levels nationwide, with β value (95%CI) being − 2.75 (-3.33, -2.17), 1.66 (0.89, 2.44), and − 2.14 (-2.78, -1.51), respectively. Further analysis stratified by *H. pylori* infection status showed that both high salt and frequent fruit intake remained significantly associated with PGR. Baseline serum PG levels, independent of gastric mucosal lesions, exhibit significant regional variations across China. These variations are primarily associated with *H. pylori* infection status and dietary factors. Our findings suggest that region-specific and *H. pylori*-stratified PG cut-off values may be warranted for gastric cancer (GC) screening, pending prospective validation.

**Supplementary Information:**

The online version contains supplementary material available at 10.1038/s41598-026-38326-9.

## Introduction

Gastric cancer (GC) is the fifth most common cancer globally, leading to unaffordable medical costs and resources challenges, as endoscopy—the gold standard for diagnosis—is frequently employed^1–3^. Correa’s cascade, also known as pathological progression from gastritis to GC, is reversible in some stages^4^, underscoring the importance of early diagnosis for improved prognosis and reduced costs.

Serum pepsinogens (PGs) serve as biomarkers for gastric mucosal health. Multiple studies have confirmed that the level of PGI and pepsinogen I/II ratio (PGR) would decline as non-atrophic gastritis (NAG) progresses to atrophic gastritis (AG), intestinal metaplasia (IM), dysplasia and ultimately GC^5–7^. Their levels are influenced by multiple factors, including acidity, dietary protein intake, hormones, age, diseases (including chronic gastritis, ulcers, gastric atrophy, or renal dysfunction), *helicobacter pylori* (*H. pylori*) infection, medications, genetic variations, stress and lifestyle^8–10^. Interregional differences are mainly reflected in variations in lifestyle and disease distribution. Multiple studies have demonstrated that PGs’ diagnostic cutoff values vary significantly across different countries or regions. In Japan, the criteria for screening individuals at high risk of GC are defined as PGI ≤ 70ng/ml and PGR ≤ 3. This screening strategy yields a sensitivity and specificity ranging from 73.9% to 77.9% and 61.8% to 72.7%^11^, respectively. But similar standards have not been established in China. Our previous research has found that the best cutoff value for GC was PGR ≤ 4.7 (AUC = 0.715) within China^5^. When the *H. pylori* infection status was taken into account, the diagnostic cutoff values for *H. pylori*-negative subgroup was PGR ≤ 7.1 (AUC = 0.797), while there was no best cutoff value for *H. pylori*-positive subgroup^12^. However, evidence is limited in whether serum PG levels exist regional heterogeneity irrespective of gastric mucosal health. Thus, recognizing the significant geographical and lifestyle variations across China, we conducted a multi-center cross-sectional study to investigate the variability of baseline serum PG levels throughout the country and identify their potential influencing factors.

## Methods

### Study design and population

The cross-sectional multi-center study used individual-level data from twelve Health Management Centers in various regions of China. These participating centers were respectively located in Southern China (No. 924 Hospital of the People’s Liberation Army of China), Eastern China (the Second Affiliated Hospital of Zhejiang University School of Medicine, Zhongshan Hospital affiliated to Xiamen University, Traditional Chinese Medicine Hospital of Kunshan, the Second Military Medical University, Ningbo No.1 Hospital, Zhejiang Provincial Hospital of Traditional Chinese Medicine), Southwest China (Sichuan Provincial People’s Hospital and the First Hospital affiliated to AMU), Northeast China (Jilin People’s Hospital), Northern China (Chinese PLA Hospital), and Central China (Jinzhou Hospital of Traditional Chinese Medicine). Individuals who underwent regular health checkup from October 2016 to October 2021 were screened in the study. Inclusion criteria were: having the intention to undergo gastroscopy during the health check-up. Exclusion criteria were: incomplete data, contraindications for gastroscopy, a history of gastrectomy or the usage of proton pump inhibitors (PPIs) within the last month, declined cognitive function.

The study was approved by the Human Research Ethics Committee of the participating hospitals (approval number: 2024 − 1181 at the Second Affiliated Hospital of Zhejiang University, the leading study site). All methods were performed in accordance with the relevant guidelines and regulations and the Declaration of Helsinki. Written informed consent was obtained from all participants.

### Questionnaire survey

Data were partly collected from participants using a self-administered questionnaire. This instrument captured baseline demographic information (age, sex, nationality), lifestyle factors including smoking (defined as consuming > 1 cigarette per day for ≥ 1 year) and alcohol consumption (> once weekly for ≥ 1 consecutive year), and dietary patterns. Dietary habits specifically assessed included the frequency of fruit, vegetable, and dairy intake (categorized as ‘frequent’ if consumed > 3 times per week, otherwise ‘occasional’) and high salt intake (> 10 g of salt per day). The questionnaire also gathered information on surgical history, current medication use, and family history of gastric cancer among first-degree relatives. Concurrently, the Mini-Mental State Examination (MMSE) was administered during clinical assessment visits to screen for cognitive function. Cognitive dysfunction was defined as an MMSE score below 27.

### Data collection

Objective data were extracted from the Hospital Information System, including gastroscopy results, pathological diagnosis, serum PG levels, 13C-urea breath test (Shenzhen Zhonghe Headway Bio-Sci & Tech Co. Ltd., China), and/or *H. pylori* serological tests (MP Biomedicals, Santa Ana, CA, USA). All test samples were collected on the same day according to the manufacturer instructions and verified by another researcher.

*H. pylori* infection status was assessed by pathological results, 13C-urea breath test, and/or serological tests. Participants were classified as *H. pylori* -positive (including prior eradication) if any available test result was positive; otherwise, they were classified as *H. pylori*-negative. Fasting blood samples (5 ml) were collected after an overnight fast and centrifuged at 10,000 g for 10 min. Serum PGI and PGII levels were measured locally at each participating center’s clinical laboratory using the chemiluminescent microparticle immunoassay method with the Abbott ARCHITECT Pepsinogen I and II Reagent Kit (Abbott Laboratories Inc., Chicago, IL, USA). Calibration and internal quality controls were performed according to the manufacturer’s recommendations and each laboratory’s standard operating procedures.

Upper gastrointestinal endoscopy and biopsies were performed by experienced endoscopists. For OLGA staging, 4–6 biopsies were typically obtained using a standardized mapping approach aligned with the Houston-updated Sydney system: at least two biopsies from the antrum (lesser and greater curvature, approximately 3 cm from the pylorus) and at least two from the corpus (lesser curvature approximately 4 cm proximal to the incisura and mid greater curvature), with a biopsy from the incisura angularis when feasible in the health-check setting; additional targeted biopsies were taken from any endoscopically visible focal lesions as clinically indicated. Specimens were submitted in separately labeled vials according to topographic site (antrum [± incisura] and corpus). Gastric atrophy severity was staged using the Operative Link on Gastritis assessment (OLGA) system (stages 0–IV), and gastric cancer (GC) was diagnosed histopathologically and reported as a separate category. Final diagnoses were reached by consensus among two or more experienced pathologists, especially for complex cases. Participants were classified into four groups: NAG (OLGA-0), MAG (OLGA-I/II), SAG (OLGA-III/IV), and GC.

### Statistical analysis

Continuous variables were tested for normality using the Kolmogorov–Smirnov test. Data with a normal distribution were represented as mean ± standard deviation (SD) and analyzed using ANOVA with post hoc Scheffé’s test. Non-normally distributed data were reported as median (interquartile range [IQR]). Categorical data were analyzed using the Chi-square test and Bonferroni post hoc test. The correlation between PGR and potential influencing factors was evaluated using multiple linear regression. And *p* value < 0.05 was considered statistically significant. Statistical analyses were performed using Stata18 for Mac (Stata-Corp, College Station, TX, USA).

## Results

### Demographical and clinical characteristics

A total of 2,902 participants were included in the study, comprising 1,248 females (43.0%) and 1,654 males (57.0%). The distribution across OLGA stages (0–IV) and gastric cancer (GC) was as follows: 2,382 (82.08%) in OLGA-0, 229 (7.90%) in OLGA-I, 137 (4.72%) in OLGA-II, 56 (1.92%) in OLGA-III, 6 (0.21%) in OLGA-IV, and 92 (3.17%) with histopathologically confirmed GC. Age exhibited an increasing trend with advancing gastric mucosal atrophy. A similar trend was observed for *H. pylori* positivity. Specifically, *H. pylori* positivity was significantly higher in OLGA stages I–IV than in OLGA-0 (*p* < 0.05) and peaked in OLGA-IV grade; however, the positivity rate in the GC group was lower than that observed in OLGA-0 (Table 1).

**Table 1:** Gender, Age, and *H. pylori* infection statu*s* across OLGA stages and gastric cancer (GC).

	OLGA-0 (*n* = 2,382)	OLGA-I (*n* = 229)	OLGA-II (*n* = 137)	OLGA-III (*n* = 56)	OLGA-IV (*n* = 6)	GC (*n* = 92)	Total (*n* = 2,902)	*p* value
Age (years,$\:\overline{x}\:$± s)	52.73 ± 10.48	55.96 ± 9.17^*^	56.29 ± 8.11^*^	54.98 ± 10.22	56.67 ± 8.80	60.41 ± 12.06^*^	53.45 ± 10.46	< 0.001
**Gender**								
female, n (%)	1,039 (43.62%)	101 (44.10%)	47 (34.31%)^*^	21 (37.50%)	2 (33.33%)	38 (41.30%)	1,248 (43.00%)	0.333
male, n (%)	1,343 (56.38%)	128 (55.90%)	90 (65.69%)	35 (62.50%)	4 (66.67%)	54 (58.70%)	1,654 (57.00%)	
***H.pylori*** **infection status**								
*H.pylori*-negative, n (%)	1,609 (67.55%)	140 (61.14%)^*^	71 (51.82%)^*^	27 (48.21%)^*^	1 (16.67%)^*^	68 (73.91%)	1,916 (66.02%)	< 0.001
*H.pylori*-positive, n (%)	773 (32.45%)	89 (38.86%)	66 (48.18%)	29 (51.79%)	5 (83.33%)	24 (26.09%)	986 (33.98%)	

^*^Compared with OLGA-0, *p* < 0.05.

### Levels of serum PGs among various pathological groups

Compared with the NAG group, both PGI and PGR levels were significantly lower and exhibited a progressive decrease from MAG to SAG group, and further declined in the GC group (*p* < 0.05). Conversely, PGII levels were elevated in the MAG, SAG, and GC groups compared to the NAG group, with statistically significant difference observed for the SAG and GC groups relative to NAG (*p* < 0.05) (Table 2).

**Table 2:** PG levels in various pathological stages.

	NAG (*n* = 2,382)	MAG (*n* = 366)	SAG (*n* = 62)	GC (*n* = 92)	Total (*n* = 2,902)	*p* value
**PGI** [ng/ml, M(IQR)]	91.79 (76.21)	73.40 (73.03)^*^	64.65 (62.50) ^*^	63.95 (59.84)^*^	88.32 (76.42)	< 0.001
**PGII** [ng/ml, M(IQR)]	8.02 (7.08)	8.20 (8.70)	10.10 (10.12)^*^	9.31 (10.16)^*^	8.13 (7.40)	< 0.001
**PGR** [M(IQR)]	10.63 (9.07)	8.41 (6.51)^*^	7.05 (6.26)^*^	7.00 (6.67)^*^	10.08 (9.01)	< 0.001

NAG non-atrophic gastritis; MAG mild-moderate atrophic gastritis; SAG severe atrophic gastritis; GC gastric cancer.

^*^Compared with NAG, *p* < 0.05.

### Baseline levels of serum PGs and potential influencing factors across China

Further analysis showed that PG levels varied among baseline populations (namely OLGA-0 grade) across different regions of China (*p* < 0.05). Notably, PGI and PGR levels in Central China were significantly different from those in other regions, with the exception of Southern China. Regional variation was also observed in factors potentially influencing PG levels. Demographic factors (age, gender, and nationality), as well as several modifiable factors including dietary habits, smoking status, drinking habits, and *Helicobacter pylori* (*H. pylori*) infection status, all showed significant differences across the six regions (*p* < 0.05). Individuals in Northeast China had the highest prevalence of high salt diets (32.5%), followed by Southern (19%) and Southwest China (18.3%). Prevalence in these regions were significantly higher than those in Eastern (12.5%), Northern (13.6%), and Central China (3.1%) (*p* < 0.05). Individuals in Northeast, Northern, and Southwest China also had a significantly higher proportion of fruit intake (61.1%, 61.1% and 60.6%, respectively) compared to Southern, Eastern, and Central China (39.4%, 43.5% and 46.9%, respectively) (*p* < 0.05). A significantly higher proportion of vegetable consumption was reported in all other regions (ranging from 80.9% to 93.8%) compared to Eastern China (68.7%) (*p* < 0.05). Conversely, lower rates of dairy intake were noted in Central China (6.2%) and Southern China (7.7%), with these proportions differing significantly from the other regions (*p* < 0.05). Smoking prevalence was significantly higher in Southern China (31.7%) than in the other regions (*p* < 0.05). Similarly, statistically higher drinking rates were found in Southern (31%), Southwest (29.7%), Northeast (28.1%), and Northern (29%) China (*p* < 0.05). Furthermore, Southern China exhibited the highest rate of *H. pylori* infection (51.4%), which was significantly greater than observed in the other regions (*p* < 0.05) (Table 3). When applying fixed PG thresholds to the baseline (OLGA-0) population, region-specific positivity rates varied substantially (Japanese criterion: 0–6.34%; China cutoff: 0–28.40%; Supplementary Table 1).

**Table 3:** Variations in baseline serum PG levels and potential influencing factors across China.

	Southern China (*n* = 142)	Eastern China (*n* = 1,668)	Southwest China (*n* = 175)	Northeast China (*n* = 203)	Northern China (*n* = 162)	Central China (*n* = 32)	*p* value
**PGI** [ng/ml, M(IQR)]	133.15(86.96)^acf^	188.43(76.95)^be^	72.12(50.69)^ce^	109.34(62.66)^df^	79.00(72.50)^e^	118.77(59.17)^f^	< 0.001
**PGII** [ng/ml, M(IQR)]	8.92(11.11)^abde^	7.80(6.50)^bf^	6.13(5.18)^cf.^	10.44(10.92)^de^	10.58(8.28)^e^	5.87(6.46)^f^	< 0.001
**PGR** [M(IQR)]	16.01 (10.62)^af^	10.39(8.62)^bcd^	11.63(7.92)^cd^	10.77(7.45)^d^	6.35(8.29)^e^	19.47(12.92)^f^	< 0.001
**age** (years,$\:\overline{x}\:$± s)	51.62 ± 8.23^abcef^	52.88 ± 10.89^bdef^	48.77 ± 9.64^c^	54.74 ± 8.25^def^	53.11 ± 10.56^ef^	56.53 ± 8.21^f^	< 0.001
**Gender**							
female, n (%)	67 (47.2%) ^abcdef^	691 (41.4%) ^bce^	79 (45.1%) ^cde^	103 (50.7%) ^def^	78 (48.1%) ^ef^	21 (65.6%) ^f^	0.007
male, n (%)	75 (52.8%)	977 (58.6%)	96 (54.9%)	100 (49.3%)	84 (51.9%)	11 (34.4%)	
**Nationality**							
Han nationality, n (%)	123 (86.6%) ^acde^	1,664 (99.8%) ^bf^	160 (91.4%) ^cdef^	180 (88.7%) ^de^	142 (87.7%) ^e^	32 (100.0%) ^f^	< 0.001
minority, n (%)	19 (13.4%)	4 (0.2%)	15 (8.6%)	23 (11.3%)	20 (11.3%)	0 (0.0%)	
**High salt diet**							
no, n (%)	115 (81.0%) ^ace^	1,459 (87.5%) ^bef^	143 (81.7%) ^ce^	137 (67.5%) ^d^	140 (86.4%) ^ef^	31 (96.9%) ^f^	< 0.001
yes, n (%)	27 (19.0%)	209 (12.5%)	32 (18.3%)	66 (32.5%)	22 (13.6%)	1 (3.1%)	
**Fruits**							
occasionally, n (%)	86 (60.6%) ^abf^	943 (56.5%) ^bf^	69 (39.4%) ^cdef^	94 (46.3%) ^def^	63 (38.9%) ^ef^	17 (53.1%) ^f^	< 0.001
frequently, n (%)	56 (39.4%)	725 (43.5%)	106 (60.6%)	109 (61.1%)	99 (61.1%)	15 (46.9%)	
**Vegetable**							
occasionally, n (%)	24 (16.9%) ^acdef^	522 (31.3%) ^b^	19 (10.6%) ^cdf^	28 (13.8%) ^def^	31 (19.1%) ^ef^	2 (6.2%) ^f^	< 0.001
frequently, n (%)	118 (83.1%)	1,146 (68.7%)	156 (89.1%)	175 (86.2%)	131 (80.9%)	30 (93.8%)	
**Dairy intake**							
occasionally, n (%)	131 (92.3%) ^af^	1,403 (84.1%) ^bf^	101 (57.7%) ^c^	144 (70.9%) ^de^	111 (68.5%) ^e^	30 (93.8%) ^f^	< 0.001
frequently, n (%)	11 (7.7%)	265 (15.9%)	74 (42.3%)	59 (29.1%)	51 (31.5%)	2 (6.2%)	
**Smoking**							
no, n (%)	97 (68.3%) ^acdf^	1,386 (83.1%) ^bef^	131 (74.9%) ^cdef^	142 (70.0%) ^df^	134 (82.7%) ^ef^	27 (84.4%) ^f^	< 0.001
yes, n (%)	45 (31.7%)	282 (16.9%)	44 (25.1%)	61 (30.0%)	28 (17.3%)	5 (15.6%)	
**Drinking**							
no, n (%)	98 (69.0%) ^acde^	1,402 (84.1%) ^bf^	123 (70.3%) ^cde^	146 (71.9%) ^def^	115 (71.0%) ^ef^	28 (87.5%) ^f^	< 0.001
yes, n (%)	44 (31.0%)	266 (15.9%)	52 (29.7%)	57 (28.1%)	47 (29.0%)	4 (12.5%)	
***H.pylori*** **infection status**							
*H.pylori*-negative, n (%)	69 (48.6%) ^a^	1,137 (68.2%) ^bcdf^	117 (66.9%) ^cd^	135 (66.5%) ^d^	124 (76.5%) ^ef^	27 (84.4%) ^f^	< 0.001
*H.pylori*-positive, n (%)	73 (51.4%)	531 (31.8%)	58 (33.1%)	68 (33.5%)	38 (23.5%)	5 (15.6%)	

^a, b, c, d, e, f^ *p >* 0.05 when groups share the same letter.

### Independent correlations between PGR and various factors

In the baseline populations (OLGA-0), multiple linear regression analysis revealed that age, gender (female as the reference), *H.pylori* infection status (*H.pylori*-negative as the reference), high salt intake, and dietary habits (vegetable and fruit intake) were independently correlated with PGR levels. The adjusted β (95%CI) were as follows: age 0.03 (0.01, 0.06), male − 0.75 (−1.36, −0.13), *H.pylori*-positive − 2.75 (−3.33, −2.17), high salt intake 1.66 (0.89, 2.44), frequent vegetable intake 1.37(0.67, 2.06) and frequent fruit intake − 2.14 (−2.78, −1.51). In subgroup analyses stratified by *H. pylori* infection status, the associations remained broadly consistent. In the *H.pylori*-positive group, β value (95%CI) for high salt intake and frequent fruit intake were 1.91 (0.67, 3.16) and − 2.30 (−3.34, −1.27), respectively. Correspondingly, the values for the *H.pylori*-negative group were 1.56 (0.58, 2.55) and − 2.02 (−2.82, −1.22) (Table 4). Sensitivity analyses using cluster-robust standard errors and excluding the smallest region yielded similar estimates (Supplementary Table 2).

**Table 4:** Correlations between baseline PGR and multiple factors according to *H. pylori* infection Status.

	OLGA0		H. pylori-positive		H. pylori-negative	
	β (95%CI)	*p* value	β (95%CI)	*p* value	β (95%CI)	*p* value
**Age** (years)	0.03(0.01,0.06)	0.011	0.01(−0.04,0.05)	0.753	0.05(0.01,0.08)	0.005
**Gender** female male	reference −0.75(−1.36,−0.13)	0.017	reference −1.32(−2.31,−0.34)	0.009	reference −0.46(−1.23,0.31)	0.241
**Nationality** Han nationality minority	reference −0.03(−1.53,1.48)	0.972	reference 1.13(−0.96,3.22)	0.288	reference −1.04(−3.12,1.05)	0.329
**High salt diet** no yes	reference 1.66(0.89,2.44)	< 0.001	reference 1.91(0.67,3.16)	0.003	reference 1.56(0.58,2.55)	0.002
**Fruits** occasionally frequently	reference −2.14(−2.78,−1.51)	< 0.001	reference −2.30(−3.34, −1.27)	< 0.001	reference −2.02(−2.82, −1.22)	< 0.001
**Vegetable** occasionally frequently	reference 1.37(0.67,2.06)	< 0.001	reference 0.87(−0.31, 2.05)	0.148	reference 1.60(0.73,2.46)	< 0.001
**Dairy intake** occasionally frequently	reference −0.08(−0.80,0.65)	0.839	reference 0.46(−0.66,1.57)	0.420	reference −0.40(−1.34,0.54)	0.401
**Smoking** no yes	reference −0.34(−1.15,0.47)	0.412	reference 0.68(−0.58,1.93)	0.290	reference −0.89(−1.94,0.15)	0.092
**Drinking** no yes	reference 0.52(−0.28,1.32)	0.203	reference 0.94(−0.30,2.18)	0.137	reference 0.33(−0.70,1.37)	0.526
***H.pylori*** **infection status** *H.pylori*-negative *H.pylori*-positive	reference −2.75(−3.33,−2.17)	< 0.001	-	-	-	-

## Discussion

Correa’s cascade describes the pathological progression from gastritis through gastric precancerous lesions, culminating in gastric cancer (GC). The transition from severe atrophic gastritis (AG) and intestinal metaplasia (IM) is considered a point of no return due to the compromised gastric epithelial barrier^4,13^. Recognizing the irreversibility of certain stages and the cost-effectiveness of serum PG testing, this study analyzed data from twelve Health Management Centers across various regions in China to investigate the regional variability in baseline serum PG levels and potential factors influencing these levels. The key findings of the study are as follows: (i) Significant regional differences in baseline serum PG levels are observed across China, with PGI and PGR levels displaying similarities in Southern and Central China; (ii) Direct association of baseline serum PGR levels are detected with *H.pylori* infection status, high salt intake, and frequent fruit intake. Additional findings include: (i) Variations in age and *H.pylori* infection status across OLGA groups, although no gender differences are detected; (ii) A trend where PGI and PGR levels decline progressively with increasing severity of gastric mucosal atrophy, whereas PGII levels tend to increase; (iii) A notable decrease in *H.pylori* infection rates as the pathological classification reaches the GC group, following a plateau observed at OLGA-IV.

GC development is characterized by various morphological and functional alterations, including disruption of the gastric mucosal barrier and diminished secretory function. Serum PGs can effectively reflect these changes. PGI is produced by chief and neck cells in the fundus and body of the stomach, while PGII is synthesized in the pyloric glands of the antrum and Brunner’s glands of the proximal duodenum^14^. As inflammation or atrophy becomes more corpus-predominant, PGI levels decrease while PGII levels increase, resulting in a lower PGR. Numerous patient-centered studies have demonstrated regional variations in PG levels, and documented that PG levels vary with the degree of gastric mucosal atrophy. Typically, PGI ≤ 70ng/ml and PGR < 3 are recommended as diagnostic cutoff values for identifying individuals at elevated GC risk in high-incidence regions such as Japan, South Korea, and China. However, some region-specific studies and analyses based on gastric mucosal state have identified variability in optimal PGI and PGR thresholds. For instance, a Japanese cohort study demonstrated that lower PGI (≤ 20.1ng/ml) and PGR (≤ 1.8) are highly effective in screening for atrophic gastritis (AG), with an area under the curve (AUC) of 0.932^7^. Our prior multicenter research determined that the diagnostic cutoff values for AG were PGI ≤ 73.1ng/ml (AUC = 0.596) and PGR ≤ 9.8 (AUC = 0.636), while the corresponding value for GC was PGR ≤ 4.7 (AUC = 0.715)^15^. Another study in Eastern China suggested a more stringent cutoff for GC, with PGI ≤ 51.2ng/ml (AUC = 0.843)^16^. Although serum PG levels among patients-focused cohorts exhibit regional variation, there is a notable lack of researches examining these levels within baseline populations. Our research addresses this gap by clarifying the regional differences in baseline serum PG levels, allowing us to conclude that regional variations in serum PG levels exist across the general population, ranging from NAG to GC.

Regional differences in GC incidence are not fully accounted for by the *H.pylori*-positive prevalence, as certain areas exhibit higher rates of *H.pylori* infection but lower GC incidence^17^. Dietary factors, including high salt intake, consumption of red or processed meats, and fried foods, are generally recognized as contributing factors to the increased incidence of AG and GC. As baseline serum PG levels serve as indicators of structural and functional changes in the gastric mucosa, our study further hypothesized and confirmed correlations between baseline serum PG levels and dietary habits. When stratified by *H.p*ylori infection status, frequent fruit consumption and high salt intake remained robustly associated with baseline serum PG levels, consistent with findings from previous studies^18–21^. A study conducted in Korea demonstrated a reduced risk of GC associated with frequent fruit consumption (OR = 0.47; 95%CI 0.28–0.77). This protective effect was potentially mediated by the modulation of gastric microbial dysbiosis^18^. Extensive research has indicated that dietary patterns influence gut inflammation, partly mediated by interactions with the gut microbiota and inflammation responses^22,23^. Furthermore, serum PGs have shown associations with interleukin-1 cytokine levels and microbiome profiles in populations with gastroesophageal reflux^24^.

*H.pylori* is established as a class I carcinogen for GC, facilitating progression along Correa’s cascade. Previous studies have documented that spontaneous *H.pylori* clearance results in a predominance of non-*H.pylori* microbiota and a subsequent reduction in microbiota diversity^25,26^. Notably, our study identified a marked reduction in *H.pylori* infection rates as pathological classifications progressed to GC group, in contrast to the highest infection rate observed at OLGA-IV grade. This observation underscores the importance of comprehensive screening and timely *H.pylori* eradication, particularly during pathologically reversible stages. Research has shown that asymptomatic *H.pylori*-positive individuals display elevated serum levels of PGI and PGII, especially PGII, resulting in a substantially reduced PGR compared to non-infected individuals^27,28^. Consistent with these findings, our study revealed that the mean PGR was lower by 2.75 in *H.pylori*-positive individuals compared to *H.pylori* -negative individuals.

The study presents several key innovations: (i) The identification of regional variations in PG levels and their associated factors offers valuable insights that can inform the development of region-specific preventions and diagnostic strategies; (ii) Focusing specifically on a population with pathologically confirmed NAG, enhances the interpretability and utility compared to patients-focused studies; (iii) Recruitment of participants from asymptomatic health checkup populations effectively minimized the risk of selection bias. However, the study is subject to certain limitations: (i) The cross-sectional design precludes the tracking of changes in PG levels over time, thereby limiting the ability to infer causality; (ii) Reliance on self-reported questionnaires for obtaining data is susceptible to recall bias, although efforts were made to mitigate this by excluding participants with cognitive impairments; (iii) The analysis was limited to dietary factors, other potentially important factors known to affect PG levels, such as socioeconomic status, environmental factors, healthcare accessibility and renal function, could not be evaluated due to missing data; (iv) Because the combinations of *H. pylori* tests (histology, 13 C-urea breath test, and serology) were not uniform across centers, and serology does not reliably indicate active infection, we could not stratify *H. pylori* status into currently infected, previously infected/eradicated, and never infected categories in the full cohort. This pragmatic definition may have introduced infection-status misclassification and attenuated between-group contrasts; (v) Serum pepsinogen assays and biopsies were performed in local laboratories at participating centers; therefore, inter-laboratory variation cannot be completely excluded and may introduce additional measurement variability; (vi) Regional sample sizes were highly imbalanced (e.g., Eastern China *n* = 1,668 vs. Central China *n* = 32), which may reduce the precision of region-specific estimates; thus, comparisons involving very small regional strata should be interpreted cautiously.

## Conclusions

In conclusion, substantial regional differences in PG levels and their determinants—particularly age, *H.pylori* infection status, and dietary habits—are observed across China. Our findings suggest that region-specific and *H. pylori*-stratified PG cut-off values may be warranted to optimize prescreening for gastric cancer and precancerous lesions, pending prospective validation.

## Supplementary Information

Below is the link to the electronic supplementary material.

Supplementary Material 1

## Data Availability

The datasets generated and analyzed during the current study are available from the corresponding author upon reasonable request.

## Abbreviations

PG: pepsinogen
PGI: pepsinogen I
PGII: pepsinogen II
PGR: pepsinogen I/II ratio
H.pylori: Helicobacter pylori
NAG: non-atrophic gastritis
GC: gastric cancer
AG: atrophic gastritis
IM: intestinal metaplasia
OLGA: the Operative Link on Gastritis assessment
MAG: mild-to-moderate atrophic gastritis
SAG: severe atrophic gastritis
IQR: interquartile range
SD: standard deviation
MMSE: Mini-Mental State Examination
PPI: proton pump inhibitor

## References

[1] Noh, C. K. et al. Association of intensive endoscopic screening burden with gastric cancer detection. *JAMA Netw. Open.* **4** (1), e2032542 (2021). 33410877 10.1001/jamanetworkopen.2020.32542 PMC7791358

[2] Suh, Y. S. et al. National cancer screening program for gastric cancer in korea: nationwide treatment benefit and cost. *Cancer* **126** (9), 1929–1939 (2020). 32031687 10.1002/cncr.32753

[3] Thrift, A. P. & El-Serag, H. B. Burden of gastric cancer. *Clin. Gastroenterol. Hepatol.* **18** (3), 534–542 (2020). 31362118 10.1016/j.cgh.2019.07.045 PMC8859863

[4] Xi, J., Li, Y., Zhang, H. & Bai, Z. Dynamic variations of the gastric microbiota: key therapeutic points in the reversal of correa’s cascade. *Int. J. Cancer*. **152** (6), 1069–1084 (2023). 36029278 10.1002/ijc.34264

[5] Fan, X. et al. Screening for gastric cancer in china: Advances, challenges and visions. *Chin. J. Cancer Res.* **33** (2), 168–180 (2021). 34158737 10.21147/j.issn.1000-9604.2021.02.05 PMC8181866

[6] Gantuya, B. et al. Evaluation of serum markers for gastric cancer and its precursor diseases among high incidence and mortality rate of gastric cancer area. *Gastric Cancer*. **22** (1), 104–112 (2019). 29934751 10.1007/s10120-018-0844-8

[7] Kishikawa, H. et al. Relevance of pepsinogen, gastrin, and endoscopic atrophy in the diagnosis of autoimmune gastritis. *Sci. Rep.* **12** (1), 4202 (2022). 35273265 10.1038/s41598-022-07947-1 PMC8913737

[8] Sun, L. P., Gong, Y. H., Wang, L. & Yuan, Y. Serum pepsinogen levels and their influencing factors: a population-based study in 6990 Chinese from North China. *World J. Gastroenterol.* **13** (48), 6562–6567 (2007). 18161928 10.3748/wjg.v13.i48.6562 PMC4611297

[9] Xu, W. et al. Sex-specific disparities of serum pepsinogen I in relation to body mass index. *Clin. Chem. Lab. Med.* **61** (11), 2010–2016 (2023). 37171227 10.1515/cclm-2023-0236

[10] Alhady, S. M. & Buttery, J. E. Plasma pepsinogen levels in three races of Malaysia. *Med. J. Aust*. **1** (5), 218–219 (1970). 4908222 10.5694/j.1326-5377.1970.tb77815.x

[11] Yoshihara, M. et al. Correlation of ratio of serum pepsinogen I and II with prevalence of gastric cancer and adenoma in Japanese subjects. *Am. J. Gastroenterol.* **93** (7), 1090–1096 (1998). 9672336 10.1111/j.1572-0241.1998.00335.x

[12] Tong, Y., Wu, Y., Song, Z., Yu, Y. & Yu, X. The potential value of serum pepsinogen for the diagnosis of atrophic gastritis among the health check-up populations in china: a diagnostic clinical research. *BMC Gastroenterol.* **17** (1), 88 (2017). 28728545 10.1186/s12876-017-0641-6 PMC5520218

[13] Wang, S. T., Yang, H. W., Zhang, W. L., Li, Z. & Ji, R. Disruption of the gastric epithelial barrier in correa’s cascade: clinical evidence via confocal endomicroscopy. *Helicobacter* **29** (2), e13065 (2024). 38443332 10.1111/hel.13065

[14] Dottori, L., Pivetta, G., Annibale, B. & Lahner, E. Update on serum biomarkers in autoimmune atrophic gastritis. *Clin. Chem.* **69** (10), 1114–1131 (2023). 37680186 10.1093/clinchem/hvad082

[15] Tong, Y. et al. Diagnostic value of serum pepsinogen levels for screening gastric cancer and atrophic gastritis in asymptomatic individuals: A Cross-Sectional study. *Front. Oncol.* **11**, 652574 (2021). 34504781 10.3389/fonc.2021.652574 PMC8421685

[16] Han, X. L. et al. Clinical value of pepsinogen in the Screening, Prevention, and diagnosis of gastric cancer. *Lab. Med.* **53** (1), 71–77 (2022). 34508270 10.1093/labmed/lmab035

[17] Herrero, R. et al. Regional variations in Helicobacter pylori infection, gastric atrophy and gastric cancer risk: the ENIGMA study in Chile. *PLoS One*. **15** (9), e0237515 (2020). 32898138 10.1371/journal.pone.0237515 PMC7478833

[18] Gunathilake, M., Lee, J. H., Choi, I. J., Kim, Y. I. & Kim, J. S. Effect of the interaction between dietary patterns and the gastric icrobiome on the risk of gastric cancer. *Nutrients*. **13**(8), 2692 (2021). 10.3390/nu13082692 PMC8401549 34444852

[19] Kim, J. et al. A comparative study of healthy dietary patterns for incident and fatal digestive system cancer. *Am. J. Gastroenterol.* **118** (11), 2061–2070 (2023). 37543749 10.14309/ajg.0000000000002448 PMC10840619

[20] Wu, X. et al. Dietary patterns and risk for gastric cancer: A case-control study in residents of the Huaihe river Basin, China. *Front. Nutr.* **10**, 1118113 (2023). 36755993 10.3389/fnut.2023.1118113 PMC9899829

[21] Gaesser, G. A., Whole & Grains Refined Grains, and cancer risk: A systematic review of Meta-Analyses of observational studies. *Nutrients*. **12**(12), 3756 (2020). 10.3390/nu12123756 PMC7762239 33297391

[22] Turpin, W. et al. Mediterranean-Like dietary pattern associations with gut Microbiome composition and subclinical Gastrointestinal inflammation. *Gastroenterology* **163** (3), 685–698 (2022). 35643175 10.1053/j.gastro.2022.05.037

[23] Ross, F. C. et al. The interplay between diet and the gut microbiome: implications for health and disease. *Nat. Rev. Microbiol.* **22** (11), 671–686 (2024). 39009882 10.1038/s41579-024-01068-4

[24] Krishnan, U., Singh, H., Tedla, N., Leach, S. T. & Kaakoush, N. O. Presence of gastric pepsinogen in the trachea is associated with altered inflammation and microbial composition. *Infect. Immun.* **88**(12), e00455-20 (2020). 10.1128/IAI.00455-20 PMC7671887 32900817

[25] Hua, Z. et al. Helicobacter pylori infection altered gastric microbiota in patients with chronic gastritis. *Front. Cell. Infect. Microbiol.* **13**, 1221433 (2023). 37662018 10.3389/fcimb.2023.1221433 PMC10470091

[26] Guo, Y. et al. Effect of Helicobacter pylori on Gastrointestinal microbiota: a population-based study in Linqu, a high-risk area of gastric cancer. *Gut* **69** (9), 1598–1607 (2020). 31857433 10.1136/gutjnl-2019-319696 PMC7456744

[27] Yu, H. et al. Serum pepsinogen II levels are doubled with Helicobacter pylori infection in an asymptomatic population of 40,383 Chinese subjects. *Med. (Baltim).* **100** (27), e26562 (2021). 10.1097/MD.0000000000026562 PMC8270603 34232200

[28] Wang, J. et al. Effects of infection with different types of Helicobacter pylori on gastric secretion function: A Cross-Sectional clinical study. *Int. J. Gen. Med.* **17**, 4539–4549 (2024). 39398484 10.2147/IJGM.S477480 PMC11471097

