# Supplementary material for: Regional variations in serum pepsinogen levels and their influencing factors: a multi-center cross-sectional study
Source: Sci Rep. 2026 Feb 8;16:7773. doi: 10.1038/s41598-026-38326-9 (PMC12948988; doi:10.1038/s41598-026-38326-9)
Supplement: Supplementary file 1 — Supplementary Material 1 [file 41598_2026_38326_MOESM1_ESM.pdf]

Regional variations in serum pepsinogen levels and their influencing factors: a multi-center cross-sectional study

HUANG Jiaojiao, YU Jiaying, TONG Yuling*

Supplementary Table 1 Positivity rates in baseline (OLGA-0) under fixed cut-offs

Region (n)	Japanese criterion, %(95CI)	China cutoff, %(95CI)
Southern China (142)	6.34% (3.33–11.74)	9.86% (5.92–15.96)
Eastern China (1,668)	1.14% (0.73–1.78)	7.25% (6.10–8.60)
Southwest China (175)	0.57% (0.08–3.95)	6.29% (3.51–11.00)
Northeast China (203)	1.48% (0.48–4.48)	7.88% (4.88–12.48)
Northern China (162)	3.70% (1.67–8.00)	28.40% (21.98–35.82)
Central China (32)	0.00% (0/32)*	0.00% (0/32)*

*No positive cases observed in Central China (n=32). Japanese criterion: PGI≤70 & PGR≤3. China cutoff PGR≤4.7.

Supplementary Table 2 Robustness analyses for Table 4 (baseline OLGA-0)

	**OLGA-0 (n=2, 382)**		**OLGA-0 (n=2, 350)**	
	**β (95% CI)**	***p* value**	**β (95% CI)**	***p* value**
**age** (years)	0.03 (-0.05, 0.12)	0.334	0.03 (-0.06, 0.13)	0.397
**gender**				
female	reference		reference	
male	-0.76 (-1.26, -0.25)	0.012	-0.65 (-1.00, -0.29)	0.007
**nationality**				
Han nationality	reference		reference	
minority	-0.04 (-2.70, 2.62)	0.972	0.04 (-2.80, 2.87)	0.973
**high salt diet**				
no	reference		reference	
yes	1.66 (-0.64, 3.95)	0.123	1.77 (-0.58, 4.11)	0.105
**fruits**				
occasionally	reference		reference	
frequently	-2.13 (-3.76, -0.51)	0.020	-2.09 (-3.98, -0.20)	0.037
**vegetable**				
occasionally	reference		reference	
frequently	1.36 (0.50, 2.23)	0.010	1.26 (0.25, 2.27)	0.026
**dairy intake**				
occasionally	reference		reference	
frequently	-0.04 (-2.10, 2.03)	0.965	0.04 (-2.12, 2.19)	0.966
**smoking**				
no	reference		reference	
yes	-0.34 (-1.22, 0.54)	0.367	-0.39 (-1.34, 0.56)	0.318
**drinking**				

	OLGA-0 (n=2, 382)		OLGA-0 (n=2, 350)	
	**β (95% CI)**	***p* value**	**β (95% CI)**	***p* value**
no	reference		reference	
yes	0.52 (-0.17, 1.22)	0.111	0.57 (-0.15, 1.29)	0.093
***H. pylori* infection status**				
H. pylori-negative	reference		reference	
H. pylori-positive	-2.76 (-3.83, -1.69)	0.001	-2.65 (-3.96, -1.34)	0.005

Model A: full sample; Model B: exclude Central China (n=32). Both models include the same covariates as Table 4 and were fitted among baseline participants (OLGA-0).
